# Do illegitimate tasks undermine employees’ pro-environmental behavior? Examining the roles of environmental concern and work overload

**DOI:** 10.3389/fpsyg.2026.1765975

**Published:** 2026-06-29

**Authors:** Abdullah Alromaihi, Muhammad Shahid Shams, Hanan Eid Badwy, Abdelaziz Ali Marzouk, Mohamed Adel Abdelrazek

**Affiliations:** 1 Department of Business Administration, College of Business and Economics, Qassim University, Buraidah, Qassim, Saudi Arabia; 2 Department of Business Administration, Abasyn University, Peshawar, Pakistan; 3 Surveying of Natural Resources in Environmental Systems Department, Environmental Studies & Research Institute, University of Sadat City, Menoufia, Egypt; 4 Business Administration Department, Faculty of Commerce, Kafr El-Sheikh University, Kafr El-Shaikh, Egypt; 5 Business Administration Department, Faculty of Commerce, University of Sadat City, Menoufia, Egypt; 6 Faculty of Media and Marketing, Midocean University, Moroni, Comoros

**Keywords:** environmental concern, higher education, illegitimate tasks, pro-environmental behavior, work overload, employment-based

## Abstract

Based on the conservation of resources theory, this study examines how illegitimate tasks relate to employees’ pro-environmental behavior through environmental concern, and how work overload shapes these relationships. Using cross-sectional survey data from 289 academic professionals in private universities in Pakistan, analyzed using partial least squares structural equation modeling (with PLS-SEM), the findings show that illegitimate tasks are negatively associated with pro-environmental behavior but positively associated with environmental concern. Moreover, environmental concern is positively associated with pro-environmental behavior. These results indicate an inconsistent mediation pattern: illegitimate tasks directly undermine pro-environmental behavior, while indirectly offsetting this negative association through heightened environmental concern. Work overload further strengthens the positive relationship between illegitimate tasks and environmental concern but weakens the relationship between environmental concern and pro-environmental behavior. The study contributes to sustainability and HRM literature by showing that employees facing illegitimate tasks may become more environmentally concerned, yet workload pressures can prevent this concern from translating into pro-environmental action. Practically, the findings suggest that reducing illegitimate tasks and managing workload are important for supporting environmentally responsible behavior in academic institutions.

## Introduction

Managers and researchers of human resource management and organizational behavior are always concerned about identifying such stressors that produce undesirable employee behaviors (Opatha, 2025). The role of tasks in shaping employees’ behavior has been a topic of interest among researchers in recent years (Bowen, 2024). Task-based stressors play a crucial role in shaping employee wellbeing and are closely associated with individuals’ perceptions of self and their work experiences (Bowen, 2024). Various studies in recent years have shed light on task-specific stressors affecting different aspects related to personnel (Ding and Kuvaas, 2023; Fila and Eatough, 2020; He et al., 2024). These stressors are linked to the level of employee outcomes in the organizational work environment; however, no study has been conducted to identify these outcomes and the procedures that lead to such outcomes in an educational institution (Ahmad et al., 2022).

Tasks are considered illegitimate when employees perceive them as unreasonable or unnecessary and inconsistent with their professional role expectations. Illegitimate tasks (ILTs), a relatively new concept, are regarded as one of the identity stressors that disrupt workers’ professional identity (Akyurek and Can, 2022). ILTs are defined as task-related stressors, tasks that breach the expectations a reasonable employee has for their job, such as tasks that are unnecessary and unreasonable (Semmer et al., 2007). Unreasonable tasks exceed the limits of the employee’s occupational role or position, potentially placing them in awkward or challenging situations. Unnecessary tasks are those that could have been avoided or are not obligatory (Ma and Xie, 2024). ILTs convey social signals of disrespect, neglect, or undervaluing, and therefore harm the employee’s self-concept (Semmer et al., 2015). Past studies considered ILTs primarily from their negative connotations. Specifically, task illegitimacy is shown to have adverse effects on workers’ emotions, work attitudes, and behavior, including self-worth, job satisfaction, emotional responses (e.g., anger), overall wellbeing, and proactive work participation (Chen et al., 2022; Wang and Jiang, 2023).

Moreover, universities worldwide are increasingly scrutinized for their environmental footprints and are expected to demonstrate responsible citizenship behavior to their students. In Pakistan, private higher-education institutions are aligning with national green campus initiatives; however, many still lag behind sector benchmarks for energy efficiency and waste minimization (Hinduja et al., 2023). Because day-to-day sustainability outcomes hinge on the discretionary actions of faculty such as switching off unused equipment, embedding environmental topics in teaching, and organizing recycling drives, it is essential to understand what motivates academic staff’s pro-environmental behaviors (PEBs) (Mendes et al., 2025).

Researchers have shown that ILTs erode employee wellbeing and suppress extra-role engagement (Mariappanadar, 2012, 2013). Assigning work that is perceived as unreasonable or unnecessary signals disrespect and increases burnout, thereby reducing employees’ willingness to participate in discretionary activities such as PEBs (Pindek et al., 2019; Zhao et al., 2023a). Conceptually, ILTs function as identity stressors that violate professional individuality (Semmer et al., 2007; Akyurek and Can, 2022). How these stressors play out in higher education, where professional identity and moral purpose are tightly bound to sustainability ideals, remains poorly understood. This micro-level focus is seen as connecting individual actions to organizational sustainability outcomes across levels (Russo et al., 2024).

To illuminate resource-draining and resource-mobilizing processes, this work draws on conservation of resources (COR) theory, which argues that individuals strive to acquire, preserve, and protect valued resources, such as time, energy, and self-esteem (Hobfoll et al., 1990). Resource loss triggered by ILTs and work overload (WOL) may depletes employees’ capacity for extra-role behavior, while environmental concern (EC) may operate as a compensatory attitudinal mechanism that partially offsets this depletion by sustaining concern for environmental issues (Vu et al., 2025; Stajkovic and Stajkovic, 2025), thereby answering recent calls to link individual action with organizational-level sustainability outcomes (Russo et al., 2024). Importantly, EC is understood here not as a resource within COR but as an attitudinal-motivational orientation—a psychological state reflecting individuals’ emotional and cognitive engagement with ecological issues—that, when sustained, can enable individuals to act on environmental values even under conditions of moderate resource strain.

However, three knowledge gaps persist. First, empirical tests linking ILTs to faculty PEBs are scarce; ILTs appear especially common in academia yet remain under-studied in that context (Ahmad et al., 2022; Ding and Kuvaas, 2023). The latest systematic review focuses almost exclusively on corporate contexts (Ding and Kuvaas, 2023). Second, no study has examined whether EC explains the complex relationship between ILTs and PEBs, including the possibility that ILTs may directly reduce behavior while indirectly sustaining it through heightened concern (Fransson and Gärling, 1999). Third, although WOL is theorized to magnify resource loss, its moderating influence on the current study’s paths remains untested in higher-education settings (Kim and Kim, 2024).

This study, therefore, asks the following question: How do ILTs influence faculty PEBs, and through which psychological mechanisms and boundary conditions do they exert this influence? The following are tested in this study (i) the direct effect of ILTs on PEBs, (ii) EC as a mediator, and (iii) WOL as a cross-path moderator. By identifying an inconsistent mediation pattern, this study shows that employees exposed to illegitimate tasks may become more environmentally concerned, yet still be less able or willing to translate this concern into pro-environmental behavior. Hence, this study makes three contributions. Theoretically, it extends COR theory to an academic-sustainability context by clarifying how resource depletion caused by ILTs influences PEBs both directly and indirectly through EC as a key psychological mechanism. Empirically, reducing ILTs and managing workload are pivotal HR levers for turning EC into observable green behavior.

## Literature review and hypothesis development

### Conservation of resources theory

**Figure 1:**
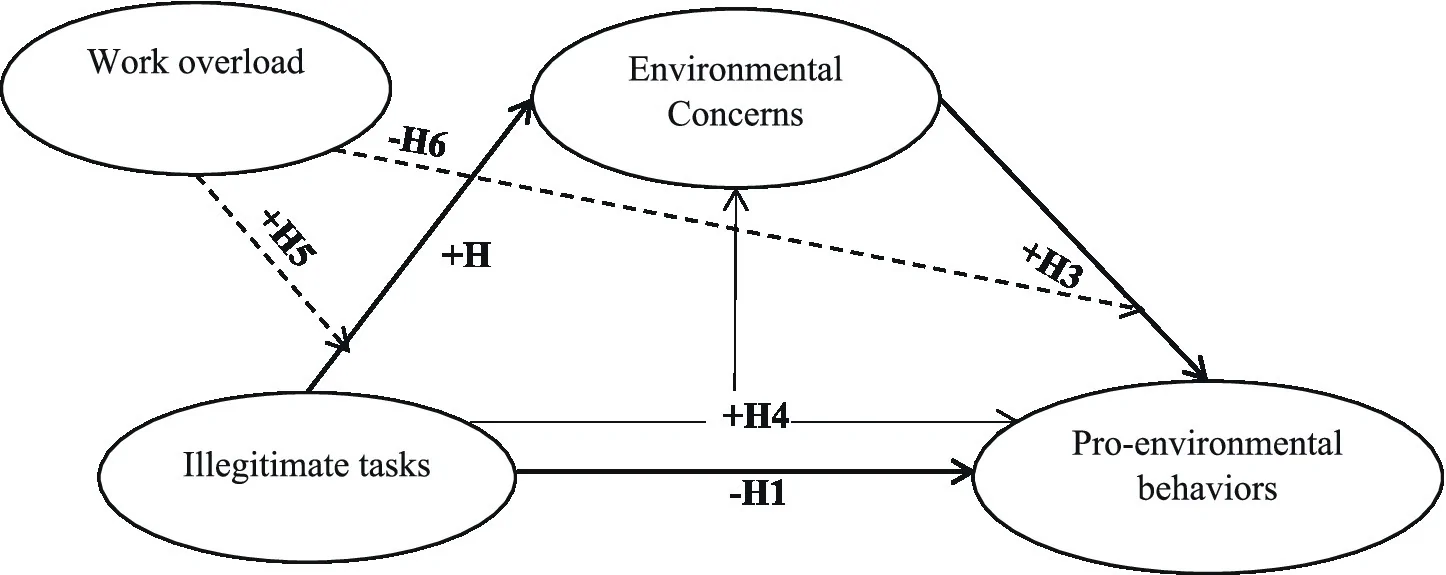
Proposed model.

Conservation of resources (COR) proposes that individuals strive to acquire, maintain, and preserve valuable resources, whether social, personal, or environmental (Hobfoll et al., 1990). In this study, COR offers a suitable lens for explaining the links between ILTs, EC, WOL, and PEBs, as illustrated in Figure 1. When workers experience ILTs, the loss of psychological or emotional resources reduces their ability or willingness to perform PEBs (Vu et al., 2025). EC, on the other hand, is conceptualized in this study as an attitudinal-motivational orientation, a psychological state reflecting individuals’ emotional and cognitive engagement with ecological issues, when sustained, EC enables individuals to enact environmental values even under stressful conditions. Importantly, environmental concern is not treated as a resource within the COR framework, but rather as a motivationally significant psychological orientation that is sensitive to resource conditions. When resource depletion is severe, this orientation may be undermined; when partially intact, it may channel residual motivation toward pro-environmental action. Conversely, WOL drains available energy and cognitive resources, weakening employees’ readiness to act on environmental issues (Stajkovic and Stajkovic, 2025). Taken together, COR theory explains how resource depletion (ILTs, overload) and resource investment (environmental concern) shape employees’ green actions, answering recent calls to bridge micro-level resource dynamics with organisational sustainability systems (Lahneman and Howard-Grenville, 2025).

## Hypothesis development

### Illegitimate tasks and pro-environmental behaviors

Employees who are assigned tasks that are perceived as illegitimate, unreasonable, and outside their official job description feel disrespected and experience a breach in their role, becoming emotionally disturbed and psychologically detached (Ouyang et al., 2022). From a COR theory perspective, such tasks constitute a direct assault on employees’ psychological resources: they drain self-esteem (a key personal resource), erode time and energy (operational resources), and signal organizational disrespect, thereby triggering a loss spiral (Hobfoll et al., 1990). Moreover, although earlier research showed the psychological cost of these tasks, there is a significant research gap as fewer studies specifically connect these HR practices to organizational sustainability results. Viewing these ILTs as ‘unsustainable’ aligns with scholarship that positions business-model design and HR architecture as levers for corporate sustainability transformation (Badwy et al., 2025a; Schaltegger et al., 2016). Employees’ intrinsic motivation and perceived value within the organization may be reduced by this perceived misalignment (i.e., their belief that assigned tasks do not fit their formal roles or responsibilities), making them less inclined to engage in optional actions such as PEBs (Badwy et al., 2025b; Kim and Kim, 2024; Pai et al., 2024).

COR theory specifically predicts that individuals facing resource loss will prioritize the conservation of remaining resources rather than investing them in discretionary activities. As a result, faculty members experiencing ILTs are expected to withdraw from non-obligatory sustainability-related behaviors in order to preserve whatever psychological resources remain available (Hobfoll et al., 1990; Zhao et al., 2023a). The literature further indicates that ILTs can elicit negative affective responses (Pindek et al., 2019) and increase burnout (Ouyang et al., 2022), depleting the psychological resources required for extra-role sustainability efforts. Zhao et al. (2023a) showed that these tasks hinder proactive behaviors, indicating that employees tend to disengage from activities not explicitly required, such as environmental sustainability practices. Accordingly, ILTs are expected to undermine employees’ PEBs by weakening the affective and motivational capacity necessary for such discretionary activities. Thus, the study suggests:

*H1:* Illegitimate tasks are negatively associated with pro-environmental behaviors.

### Illegitimate tasks and environmental concern

Illegitimate tasks are those that are considered degrading or humiliating and trigger negative emotional responses such as frustration, disengagement, and burnout (Ma and Xie, 2024). They are also perceived as unreasonable or unnecessary demands that fall outside employees’ role expectations and may threaten their professional identity (Pindek et al., 2019). Prior research has consistently shown that such tasks trigger negative emotional responses, including frustration, disengagement, and strain (Ma and Xie, 2024; Zhou et al., 2020). However, beyond their detrimental effects, illegitimate tasks may also shape how employees cognitively interpret their organizational environment.

In academic settings, particularly within private universities, the assignment of tasks that are misaligned with core teaching and research responsibilities may signal inefficiencies and a lack of institutional responsibility (Russell, 2024; Siekkinen, 2019). Such experiences can heighten employees’ awareness of broader organizational and societal issues, including environmental sustainability (Whitsed et al., 2025). When employees perceive that organizational practices involve waste, mismanagement, or disregard for meaningful work, they may become more sensitive to issues related to responsibility and sustainability (Xing and Mohamed Zainal, 2024; Yang et al., 2025).

From a COR perspective, exposure to resource-draining conditions such as illegitimate tasks may prompt employees to cognitively reappraise their environment and place greater emphasis on values that restore meaning and purpose. Environmental concern, as a value-driven and attitudinal construct, may therefore increase as employees become more aware of inefficiencies and responsibility gaps within their organizations. Accordingly, illegitimate tasks may not reduce environmental concern, but instead heighten employees’ concern for environmental issues.

*H2:* Illegitimate tasks are positively associated with environmental concern.

### Environmental concern and pro-environmental behaviors

Environmental concern refer to a person’s consciousness and emotional response to ecological problems, including but not limited to global warming, pollution, and resource depletion (Fransson and Gärling, 1999). Supposing that people, especially those in positions of authority within organizations, exhibit a high level of environmental concern, in that case, they are likely to engage in activities that demonstrate a commitment to ecological sustainability (Badwy et al., 2026; El-Tahhan et al., 2025). However, previous research indicates that there may be an intention-activity gap because organizational and contextual factors can either encourage or inhibit pro-environmental behavior, indicating that concern alone may not necessarily convert into action (Tam and Chan, 2017). Such green practices may involve energy-saving measures, recycling, waste minimization, and advocacy of green policies within one’s line of work. Within the context of higher learning institutions, faculty members who tend to express greater concern for ecological degradation are more likely to adopt sustainability principles in their instructional practices, research activities, and campus activities (Abo-Khalil, 2024). Empirical studies have demonstrated that environmental awareness serves as a motivational impulse, prompting individuals to engage in voluntary behaviors that yield ecological benefits (Hayat et al., 2025). Greater awareness and emotional attachment to ecological matters foster a propensity toward more frequent and meaningful green conduct (Hassan et al., 2024). Considering these results, EC can be seen as a major psychological stimulus that converts awareness into action; nevertheless, its efficacy is contingent upon the alignment of personal values with organizational context. Within the COR framework, this relationship reflects the principle that individuals are more likely to invest their available motivational energy in behaviors that align with their deeply held values and attitudinal commitments. When EC is intact, it provides a directional orientation for behavioral investment; the strength of this behavioral expression, however, is contingent upon the availability of sufficient residual resources to act upon that orientation (Hobfoll et al., 1990; Vu et al., 2025). This emphasizes how significant it is to incorporate contextual and individual-level elements when analyzing PEBs. It is hence theorized that:

*H3:* Environmental concern is positively associated with pro-environmental behaviors.

### Environmental concern as a mediator

Exposure to inappropriate tasks, such as excessive or irrelevant duties, can erode employees’ psychological resources and reduce their ability to engage in discretionary work behaviors (Ahmad et al., 2022). Pro-environmental behavior is one such discretionary behavior, as it often requires time, attention, and motivational investment beyond formal job requirements (Adeel et al., 2024; Chen et al., 2022). At the same time, environmental concern represents a cognitive-affective orientation that can be shaped by workplace experiences and subsequently affects behavioral responses (Lian and Ding, 2025; Lijing et al., 2022). As argued above, illegitimate tasks may heighten environmental concern by making inefficiency, waste, and responsibility gaps more salient. Environmental concern, in turn, can encourage employees’ engagement in PEBs (Tandon et al., 2024).

Therefore, environmental concern may explain an inconsistent mediation process in the relationship between ILTs and PEBs. ILTs may directly reduce PEBs through resource depletion, consistent with COR theory’s resource loss principle (Hobfoll et al., 1990). However, ILTs may also indirectly support PEBs by increasing employees’ environmental concern. Thus, environmental concern may partially counterbalance, rather than transmit, the negative relationship between ILTs and PEBs.

*H4:* Environmental concern acts as a mediator in the relationship between illegitimate tasks and pro-environmental behaviors.

### Work overload as a moderator

Conservation of resources theory offers a clear foundation for understanding how WOL intensifies the consequences of ILTs. COR posits that resource threats and losses are not simply additive but can produce compounding loss spirals when multiple stressors co-occur (Hobfoll et al., 1990). When employees simultaneously face ILTs—which deplete self-esteem, role-identity resources, and emotional energy—and high WOL—which consumes time and cognitive capacity—the cumulative resource depletion is more severe than either stressor would generate alone. When work overload is high, the experience of illegitimate tasks may become more salient and psychologically intrusive. Under such conditions, employees may become more aware of inefficiency, resource misuse, and responsibility gaps within the organization. Rather than reducing environmental concern, elevated workload may intensify the cognitive appraisal of illegitimate tasks, thereby strengthening the positive association between ILTs and EC.

Excessive professional demands stemming from exposure to ILTs can variably affect employees’ EC contingent upon the degree of WOL encountered (Ma and Peng, 2019). Elevated levels of WOL may deplete employees’ cognitive and emotional resources and intensify perceptions of strain associated with illegitimate tasks (Kim and Kim, 2024; Qalati et al., 2026). Under such conditions, employees may become increasingly attentive to organizational inefficiencies, resource misuse, and broader responsibility-related concerns. Rather than diminishing environmental concern, heightened workload may amplify employees’ cognitive appraisal of unsustainable organizational conditions, thereby strengthening the positive association between ILTs and EC. At the same time, although employees may remain environmentally concerned, excessive workload can constrain their capacity to translate such concern into concrete pro-environmental actions (Pluta and Rudawska, 2021). Accordingly, WOL serves as an important boundary condition shaping how employees cognitively and behaviorally respond to illegitimate tasks. Consequently, the study forecasts:

*H5:* Work overload moderates the relationship between illegitimate tasks and environmental concern, such that the positive relationship is stronger when work overload is high.

In the workplace, an important contextual factors is WOL, which is defined as the sensation of being overwhelmed with too many responsibilities or tasks to be accomplished within a specific period of time (Gryna, 2003). Excessive work can generate psychological stress, thereby depleting employees’ affective and cognitive resources necessary for engaging in voluntary, sustainability-oriented behaviors (Al-Romeedy and Badwy, 2025; Rupčić, 2023). Moreover, unnecessary tasks increase the burden on employees and reduce their productivity, ultimately hindering their overall performance (Zhao et al., 2023b). Although employees may have substantial environmental concern, situational constraints like workload pressure may prevent these concerns from being translated into actual behavior. Even when individuals possess a strong commitment to environmental issues, heavy workloads may undermine their abilities to initiate or participate in pro-environmental activities (Benedict, 2023). COR theory explains this dynamic through the concept of resource investment: translating attitudinal concern into active PEBs requires the investment of residual resources—time, energy, and cognitive attention. When WOL has already depleted these resources, employees lack the capacity to convert their EC into observable action, even if that concern remains psychologically present (Hobfoll et al., 1990).

For instance, stressed workers may prioritize core work tasks over behaviors such as energy conservation or promoting environmental sustainability because their focused attention is directed toward fulfilling the fundamental job requirements (Semmer et al., 2015; Szostek, 2021). This suggests that WOL negatively moderates the relationship between environmental concern and pro-environmental behavior by restricting the behavioral expression of environmental values. Therefore, WOL can be viewed as a boundary condition that limits the behavioral application of environmental values when resources are scarce, hence reducing the beneficial relationship between environmental concern and pro-environmental activity. Accordingly, the study hypothesizes:

*H6:* Work overload weakens the positive relationship between environmental concern and pro-environmental behaviors.

## Methodology

### Research design

This study employed a quantitative, cross-sectional survey design to investigate how illegitimate tasks affect PEBs among faculty members, with EC as a mediator and WOL as a moderator. The research design aligns with previous studies that have examined behavioral and attitudinal outcomes in organizational contexts using structural equation modeling (Ahmad et al., 2022; Vu et al., 2025).

### Study context and sampling

Data were collected from private sector higher education institutions in Khyber Pakhtunkhwa (KPK), Pakistan, a region experiencing growth in private higher education and evolving challenges in academic human resource practices. The region was selected due to the increasing emphasis on environmental sustainability in education and ongoing pressures on faculty arising from HR-related constraints. KPK faces pronounced environmental pressures, including recurring floods, droughts, and climate-induced resource scarcity documented in the KP Climate Change Action Plan (2022), making sustainability a salient and urgent concern for faculty, and rendering it an ecologically relevant context for studying pro-environmental behavior (Hinduja et al., 2023). Ethical approval for this study was obtained from the Institutional Ethics Review Committee (IERC) of Abasyn University, Khyber Pakhtunkhwa, Pakistan. All participants provided written informed consent before completing the survey.

Private universities were specifically targeted because prior research on unsustainable HRM practices among faculty in Pakistani private universities has established that illegitimate task assignment is a documented and detectable phenomenon in this context (Ahmad et al., 2022). Private institutions in Pakistan operate with greater managerial discretion in task allocation and less standardized HR governance than public universities, creating conditions where faculty exposure to ILTs is more variable and pronounced (Hinduja et al., 2023). Moreover, recent evidence confirms that job demands and workload significantly impair the psychological wellbeing and performance of faculty in Pakistani private universities, further establishing this sector as an ecologically valid and theoretically appropriate setting for the present investigation (Yousaf et al., 2025).

A convenience sampling method was used to recruit permanent full-time faculty members across various academic departments. Participants were eligible for inclusion if they were: (1) permanently employed full-time academics, and (2) actively engaged in teaching duties at a private university in KPK. Part-time, visiting, and contract faculty were excluded because their employment conditions and levels of HRM exposure differ substantially from permanent staff, which could introduce construct-irrelevant variance into the focal relationships (Sekaran and Bougie, 2016). Following the approach of Ahmad et al. (2022), senior administrative personnel (e.g., deans, registrars, and vice chancellors) were excluded from the study, as the constructs under investigation—such as illegitimate tasks and workload—are more directly experienced by academic teaching staff. A total of 550 questionnaires were distributed, with 320 returned (58.2% response rate). After screening, 289 valid responses were retained (52.5% effective response rate). This is sufficient according to Kline’s (2015) rule (27 items × 10 = 270).

Prior to full-scale data collection, face validity of the survey instrument was established through a panel of five subject-matter experts in HRM and organizational behavior, who reviewed all scale items for semantic clarity, contextual appropriateness, and relevance to the Pakistani private university setting. Items were retained if at least 80% of panelists confirmed their suitability, and minor wording adjustments were made to adapt internationally validated scales to the academic work context (Sekaran and Bougie, 2016). The use of previously validated scales with well-established psychometric properties further provided additional content validity assurance prior to contextual adaptation (Semmer et al., 2007; Zhang and Huang, 2019; Vu et al., 2025).

### Data collection procedure

Data were collected from August to November 2024 at a single point in time using a self-administered questionnaire, delivered in both online and paper-based formats, depending on institutional accessibility. Participants were assured of anonymity and confidentiality, and informed consent was obtained before participation. This design enabled efficient data collection while minimizing disruptions to academic schedules.

### Measures

Previously-validated multi-item scales rated on a five-point Likert scale (1 = Strongly Disagree, 5 = Strongly Agree) were used to measure the constructs in this study.

*Illegitimate tasks* were assessed using eight items from the Bern Illegitimate Tasks Scale (Semmer et al., 2007). for example: “I am frequently assigned tasks that should be done by someone else.” Semmer et al. (2007) reported reliability of *α* = 0.79–0.88, two-factor structure validity, convergent validity with work stressors (r = 0.42, *p <* 0.01), and predictive validity for burnout (*β* = 0.31, *p <* 0.001). The PLS-SEM analysis in the current study demonstrated convergent validity (AVE = 0.724) and discriminant validity (HTMT < 0.85), with an *α* value of 0.945.

*Environmental concern* was assessed using six items from Zhang and Huang (2019), for example: “I am concerned about environmental problems caused by human activities.” Zhang and Huang (2019) reported an *α* of 0.89, demonstrating convergent validity with autonomous motivation (r = 0.54, *p <* 0.01) and predictive validity for PEBs (*β* = 0.52, *p <* 0.001). The PLS-SEM analysis in the current study confirmed convergent validity (AVE = 0.708) and discriminant validity (HTMT < 0.85), with *α* = 0.918.

*Work Overload* was measured using four items from Vu et al. (2025), for example: “I have more work than I can handle during my regular working hours.” Vu et al. (2025) reported an *α* of 0.88, demonstrating convergent validity with job demands (r = 0.67, *p <* 0.01) and predictive validity for strain (*β* = 0.45, *p <* 0.001). In the current study, *α* = 0.914.

*Pro-environmental behavior* was measured using nine items from Zhang and Huang (2019). Sample item: “I encourage colleagues to adopt environmentally friendly practices at work.” Zhang and Huang (2019) reported an *α* of 0.91, demonstrating convergent validity with environmental concern (r = 0.64, *p <* 0.01) and criterion validity, which explained 58% of the variance in environmental performance. In the current study, *α* = 0.882.

All scales were contextually adapted for academic faculty in Pakistani private universities.

### Data analysis

The study’s proposed model was tested by structural equation modeling (SEM) using SmartPLS version 4.1.1 (Ringle et al., 2024). PLS-SEM was preferred because it can handle complex models with multiple mediators and moderators, it can be used for prediction-oriented analysis, and it is not sensitive to non-normality of data distributions (Hair et al., 2019). Smart PLS-SEM, in comparison to covariance-based SEM, is more flexible with smaller sample sizes and does not require multivariate normality, thus making it a good fit for the cross-sectional, survey-based design of this study. To evaluate reliability and validity in the measurement model, the two-step approach recommended by Anderson and Gerbing (1988) was followed. Next, the structural model was examined using bootstrapping with 5,000 resamples to test the hypothesized relationships. Preliminary descriptive and demographic analyses were also conducted using IBM SPSS version 23.

### Demographic profile

Table 1 presents the demographic profile of the 289 respondents. The sample was predominantly male (93.4%), with the largest age group being 31–40 years (36.3%). The majority held MPhil or PhD degrees, and most respondents were lecturers or assistant professors. The demographic characteristics reflect the academic staffing patterns commonly found in private HEIs in Pakistan.

**Table 1:** Demographic profile.

Variable	Category	Responses (289)	Percentage (%)
Gender	Male	270	93.4%
	Female	19	6.6%
Age	21–30 years	45	15.6%
	31–40 years	105	36.3%
	41–50 years	95	32.9%
	51 + years	44	15.2%
Years of Experience	< 5 years	40	13.8%
	5–10 years	105	36.3%
	11–15 years	90	31.1%
	> 15 years	54	18.7%
Qualification	Master’s	80	27.7%
	MPhil	130	45.0%
	PhD	79	27.3%
Academic Role	Lecturer	120	41.5%
	Assistant Professor	95	32.9%
	Associate Professor	60	20.8%
	Professor	14	4.8%

### Common method bias and normality testing

To address common method bias (CMB), the full collinearity test suggested by Kock and Lynn (2012) was applied, which involves regressing each latent construct on a common variable. All variance inflation factor (VIF) values were found to be below the threshold of 3.3 (see Table 2), suggesting that CMB bias is unlikely to be a concern in this dataset.

**Table 2:** Collinearity statistics.

PEB	ILTs	EC	WOL
1.256	1.482	1.519	1.318

PEB, pro-environmental behavior, ILTs, illegitimate tasks; EC, environmental concern; WOL, work overload.

In addition, data normality was assessed using Mardia’s multivariate skewness and kurtosis via the Web Power tool. The results indicated significant multivariate non-normality (Skewness *β* = 14.96083, *p <* 0.01; Kurtosis *β* = 52.07628, *p <* 0.01), further supporting the appropriateness of using PLS-SEM.

### Measurement model

The measurement model was evaluated to ensure it met the standards for convergent and discriminant validity (see Table 3). Most item loadings exceeded the recommended threshold of 0.70, and all constructs showed satisfactory values for average variance extracted (AVE > 0.50) and composite reliability (CR > 0.70), confirming convergent validity as recommended by Hair et al. (2019). In addition, the collinearity statistics for individual construct indicators were assessed using VIF values. All values ranged from 1.299 to 4.020, well below the threshold of 5.0 (Hair et al., 2019).

**Table 3:** Measurement model.

Convergent validity					
Latent variable	Items	Factor loadings	AVE	CR	VIF
Pro-environmental behaviors			0.534	0.919	
I turn off lights, computers, and equipment in my office and classroom when they are not in use.	PEB 1	0.879			3.908
I use both sides of paper and minimize printing to reduce paper waste at my university.	PEB 2	0.867			3.261
I sort waste and recycle materials in my office and academic workspace.	PEB 3	0.813			2.381
I choose environmentally friendly products and materials when making work-related purchases for my academic activities.	PEB 4	0.756			1.852
I encourage my colleagues and students to adopt environmentally friendly practices on campus.	PEB 5	0.816			2.471
I try to learn more about environmental issues relevant to higher education institutions and university campuses.	PEB 6	0.417*			1.299
I openly express my concerns about environmental challenges to colleagues and university administration.	PEB7	0.492*			1.348
I actively participate in university-level environmental sustainability initiatives and green campus programs.	PEB8	0.832			2.489
I engage in campus-based initiatives aimed at reducing the environmental footprint of my university.	PEB9	0.529*			1.426
Illegitimate tasks			0.724	0.948	
I am frequently assigned administrative tasks that should be handled by support staff, not academic faculty.	ILTs 1	0.804			2.451
I am required to perform non-teaching duties that seem unnecessary for someone in my academic role.	ILTs 2	0.818			2.849
I am given tasks that are clearly outside the scope of my teaching and research responsibilities.	ILTs 3	0.889			4.020
I have to carry out academic administrative work that makes no sense given my professional qualifications.	ILTs 4	0.855			3.079
I am asked to perform clerical or bureaucratic tasks that are beneath my level of academic expertise.	ILTs 5	0.889			3.634
I am required to complete tasks that conflict with my identity and values as an academic professional.	ILTs 6	0.874			3.602
I must fulfil assignments that are not aligned with my role expectations as a faculty member.	ILTs 7	0.838			2.739
I am delegated work by university management that has no clear academic purpose or benefit to my department.	ILTs 8	0.836			3.012
Environmental concern			0.708	0.920	
I am concerned about environmental problems caused by the activities of higher education institutions.	EC1	0.843			2.533
I feel worried about the deteriorating natural environment surrounding our university campus.	EC2	0.856			2.750
I believe environmental pollution is a serious issue that universities must urgently address.	EC3	0.877			3.503
As an academic, I feel a professional responsibility to help protect the natural environment.	EC4	0.840			2.644
I am troubled by the negative environmental impact of unsustainable practices within my university.	EC5	0.823			2.202
I believe it is important for university faculty to take action to preserve the environment for future generations.	EC6	0.809			2.114
Work Overload			0.795	0.918	
I have more teaching and administrative work than I can handle during my regular working hours at the university.	WOL 1	0.867			
My academic workload is so heavy that it is difficult to fulfill all my teaching, research, and service responsibilities on time.	WOL 2	0.909			
I feel overwhelmed by the volume of tasks assigned to me as a faculty member at this university.	WOL 3	0.872			
The academic and administrative demands placed on me by the university exceed the time and energy I have available.	WOL 4	0.918			

*Items retained despite loadings below the 0.70 threshold (Hair et al., 2019) due to their theoretical relevance to pro-environmental engagement in academic contexts.

PEB, pro-Environmental behavior; ILTs, illegitimate tasks; EC, environmental concern; WOL, work overload; AVE, average variance extracted; CR, composite reliability.

During the assessment, three items from the PEB construct, specifically, PEB6, PEB7, and PEB9 were observed to demonstrate loadings below 0.50. These items reflected faculty members’ efforts to learn more about environmental issues at work, express concerns about environmental challenges to colleagues, and participate in workplace initiatives aimed at reducing environmental impact. Although these items did not provide strong contribution statistically, they were retained because they captured meaningful behavioral expressions of environmental engagement in academic contexts; this decision is consistent with the recommendations to consider theoretical significance alongside statistical criteria (Hair et al., 2019). It is believed that these statements represent subtler but essential forms of faculty involvement with sustainability, behaviors that may not be immediately visible but are critical in shaping a long-term environmental culture within institutions.

Subsequently, the Heterotrait-Monotrait (HTMT) ratio of correlations was used to assess discriminant validity, following the guidelines of Henseler et al. (2015). All HTMT values were below the recommended threshold of 0.85 (see Table 4), providing confidence that the constructs in this model are conceptually and empirically distinct.

**Table 4:** Discriminant validity- Hetrotrait-Monotrait (HTMT) ratio.

Constructs	Mean	Std. Deviation	EC	PEB	ILTs	WOL
EC	4.051	0.231				
PEB	3.985	0.199	0.248			
ILTs	4.153	0.324	0.506	0.368		
WOL	3.508	0.569	0.378	0.382	0.032	

PEB, pro-environmental behavior; ILTs, illegitimate tasks; EC, environmental concern; WOL, work overload.

The descriptive statistics in Table 4 indicate that participants reported high levels of ILTs (M = 4.153, SD = 0.324), EC (M = 4.051, SD = 0.231), and PEB (M = 3.985, SD = 0.199), with all means exceeding the midpoint of the five-point scale. In particular, the low standard deviations for EC and PEB suggested strong agreement among respondents, whereas WOL was rated moderately high (M = 3.508, SD = 0.569), reflecting more variance in the perceived intensity of workload among faculty members across institutions.

### Structural model

Following the recommendations of Hair et al. (2019), a bootstrapping procedure was applied with 5,000 subsamples to test the significance of the proposed hypotheses. In line with the approach suggested by Hahn and Ang (2017), the importance of path coefficients was evaluated using *p*-values, confidence intervals, t-values, and effect sizes (f^2^). The results are presented in Table 5.

**Table 5:** Hypotheses testing.

Hypothesis	Relationships	Path coefficient (*β*)	Standard error (SE)	Confidence interval bias corrected (5% lower bound)	Confidence interval bias corrected (95% upper bound)	*t*-value	*p*-value	Effect size f^2^	Decision
H1	ILTs → PEB	−0.198	0.099	−0.358	−0.030	1.990	0.023	0.165	Supported
H2	ILTs → EC	0.457	0.054	0.369	0.547	8.460	< 0.001	0.468	Supported
H3	EC → PEB	0.402	0.111	0.223	0.587	3.619	< 0.001	0.096	Supported
H4	ILTs→ EC → PEB	0.184	0.056	0.097	0.278	3.282	0.001	–	Supported
H5	WOL*ILTs→EC	0.485	0.062	0.492	0.399	7.877	< 0.001	0.466	Supported
H6	WOL*EC → PEB	−0.368	0.118	−0.354	−0.537	3.134	0.001	0.135	Supported

PEB, pro-environmental behavior; ILTs, illegitimate tasks; EC, environmental concern; WOL, work overload.

The analysis revealed that ILTs had a significant negative effect on PEB (*β* = −0.198, t = 1.990, *p* = 0.023, f^2^ = 0.165), thereby supporting H1. Furthermore, ILTs had a significant positive influence on EC (*β* = 0.457, t = 8.460, *p <* 0.001, f^2^ = 0.468), thereby supporting H2. With respect to H3, EC significantly influenced PEB (*β* = 0.402, t = 3.619, *p <* 0.001, f^2^ = 0.096), providing support to H3.

The mediating role of EC in the relationship between ILTs and PEB was also examined. The results confirmed a significant positive indirect effect of ILTs on PEB through EC (*β* = 0.184, t = 3.282, *p* = 0.001), thereby supporting H4. Because the direct effect of ILTs on PEB was negative while the indirect effect through EC was positive, the findings indicate an inconsistent mediation, also referred to as a suppression pattern, in which the direct and indirect effects operate in opposite directions. This suggests that EC partially offsets, rather than simply transmit, the negative association between ILTs and employees’ pro-environmental behavior.

Regarding moderation, the interactive effects of WOL was tested on two key relationships. First, it was found that WOL significantly moderated the relationship between ILTs and EC (*β* = 0.485, t = 7.877, *p* = 0.000, f^2^ = 0.466), thereby supporting H5. Second, WOL also significantly moderated the relationship between EC and PEB, although the effect was negative (β = −0.368, t = 3.134, p = 0.001, f2 = 0.135), supporting H6.

These findings suggest that while ILTs can enhance EC, its influence becomes more pronounced under conditions of high WOL. Conversely, the ability of EC to translate into actual PEB may diminish under excessive workload, highlighting the importance of workload as a contextual barrier to sustainable engagement.

**Figure 2:**
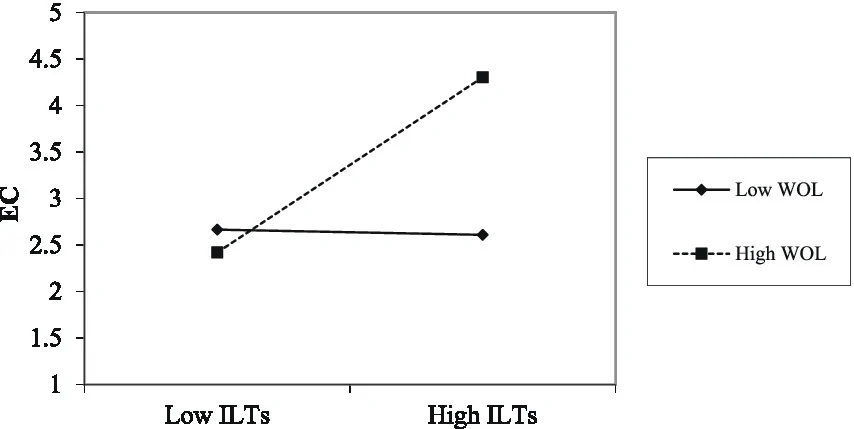
Interaction plot. ILTs, illegitimate tasks; EC, environmental concern; WOL, work overload.

**Figure 3:**
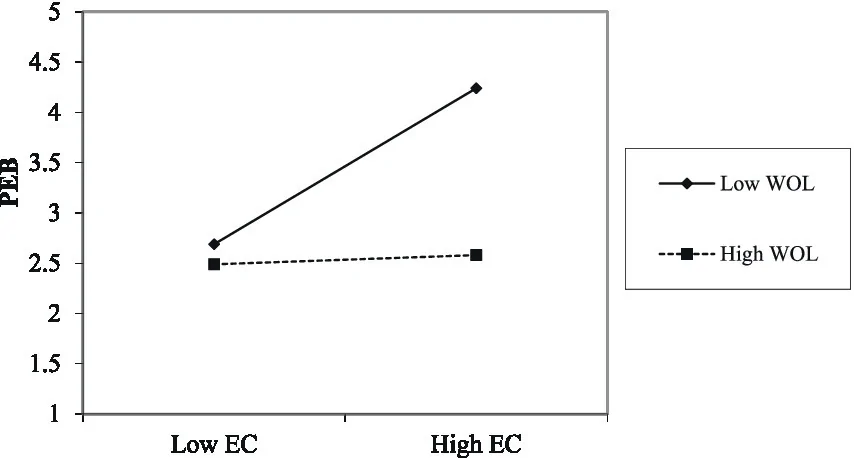
Interaction plot. EC, environmental concern; WOL, work overload; PEB, pro-environmental behavior.

To further explore the moderating influence of WOL, Dawson’s (2014) recommendation was followed and illustrated the interaction effects in Figures 2, 3. The interaction plot in Figure 2 shows that the positive relationship between ILTs and EC becomes notably stronger under conditions of high WOL. In this scenario, faculty exposed to higher levels of ILTs reported greater concern for environmental issues, particularly when WOL was high. In contrast, the relationship between ILTs and EC remained relatively flat under low WOL. This pattern suggests that WOL may intensify employees’ awareness of environmental challenges, possibly responding to broader organizational strain.

Figure 3 displays the interaction effect between EC and WOL in predicting PEBs. The plot illustrates that the positive relationship between EC and PEBs is considerably weaker under high workload conditions. When WOL is low, EC more strongly translates into environmentally responsible behaviors. This implies that excessive workload can suppress the behavioral expression of EC, highlighting the role of organizational context in enabling or hindering faculty engagement in sustainability-oriented actions.

The coefficient of determination (R^2^) values was examined to assess the model’s explanatory power. The model accounted for 56.4% of the variance in EC and 34.6% of the variance in PEBs. According to Hair et al. (2019), these values reflect moderate to substantial explanatory power, indicating that the proposed predictors explain a meaningful portion of both constructs. The higher R^2^ for EC compared to PEBs may be attributed to the more direct cognitive impact of ILTs and workload on concern formation, whereas PEBs may involve broader behavioral and contextual influences.

Finally, as recommended by Shmueli et al. (2019), PLSpredict was performed to assess the predictive power of the structural model. The Q^2^predict values demonstrate positive predictive relevance for all constructs, ranging from 0.010 to 0.441. EC indicators show medium to high predictive relevance (Q^2^predict = 0.300–0.441), while PEB indicators reflect small to medium predictive relevance (Q^2^predict = 0.010–0.214). Most indicators show lower or comparable RMSE and MAE values for the PLS-SEM model relative to the linear regression benchmark (LM), confirming that the proposed model demonstrates adequate predictive accuracy (see Table 6).

**Table 6:** PLS-predict assessment results.

Item	Q^2^ predict	PLS-SEM		LM		PLS − LM	
		RMSE	MAE	RMSE	MAE	RMSE	MAE
EC1	0.441	0.211	0.097	0.246	0.156	−0.035	−0.059
EC2	0.410	0.213	0.097	0.254	0.155	−0.041	−0.058
EC3	0.300	0.239	0.111	0.265	0.163	−0.026	−0.052
EC4	0.324	0.218	0.098	0.238	0.149	−0.020	−0.051
EC5	0.411	0.220	0.101	0.253	0.164	−0.033	−0.063
EC6	0.321	0.209	0.092	0.228	0.140	−0.019	−0.048
PEB1	0.126	0.205	0.109	0.209	0.121	−0.004	−0.012
PEB2	0.173	0.199	0.106	0.207	0.118	−0.008	−0.012
PEB3	0.129	0.204	0.106	0.210	0.112	−0.006	−0.006
PEB4	0.162	0.206	0.106	0.210	0.109	−0.004	−0.003
PEB5	0.173	0.212	0.113	0.218	0.125	−0.006	−0.012
PEB6	0.040	0.407	0.251	0.419	0.256	−0.012	−0.005
PEB7	0.010	0.441	0.253	0.455	0.258	−0.014	−0.005
PEB8	0.214	0.202	0.107	0.210	0.127	−0.008	−0.020
PEB9	0.052	0.441	0.249	0.445	0.268	−0.004	−0.019

LM, linear model; RMSE, root mean square error; MAE, mean absolute error. Negative PLS − LM values indicate the PLS-SEM model outperforms the linear benchmark. *PEB6, PEB7, and PEB9 returned low Q^2^predict values, consistent with their low factor loadings reported in the measurement model; all values remain above zero, confirming positive predictive relevance.

## Discussion

This study examined how ILTs influence PEBs among faculty in private higher education institutions in Pakistan, using EC as a mediator and WOL as a moderator. Grounded in the COR theory (Hobfoll et al., 1990), the findings reveal nuanced pathways that enrich the understanding of organizational sustainability dynamics in academic settings.

Consistent with COR theory’s core proposition, that resource depletion undermines employees’ discretionary capacities, ILTs negatively influenced PEB (H1: *β* = −0.198, *p* = 0.023). This aligns with prior research indicating that ILTs and role stressors erode psychological resources, increasing burnout and reducing engagement in voluntary behaviors such as sustainability initiatives (Pindek et al., 2019; Ouyang et al., 2022). Moreover, ILTs can foster a climate of organizational injustice, where faculty may feel undervalued or disrespected, leading them to disengage from extra-role contributions such as ecological initiatives.

The positive relationship between ILTs and EC (H2: *β* = 0.457, *p <* 0.001) suggests that faculty members experiencing ILTs exhibit elevated levels of EC. This observation aligns with previous studies indicating that employees may react to perceived discrepancies in role expectations and inequitable work assignments not solely through strain-induced responses but also via cognitive and value-oriented adaptation mechanisms (Byun et al., 2026; Cheng et al., 2025). Specifically, when individuals confront tasks perceived as extraneous to their legitimate professional obligations, they may engage in processes of meaning-making and cognitive reappraisal that enhance the prominence of alternative, socially favorable values. Within academic environments, where professional identity is profoundly intertwined with ethical accountability and societal contribution, such cognitive processes may amplify concern for broader societal challenges, including environmental sustainability (Marchetti et al., 2025; Pach et al., 2025). Consequently, ILTs may correlate with heightened EC, as faculty members reinforce prosocial and value-consistent cognitions in reaction to perceived discordance in their professional roles.

As expected, EC positively predicted PEB (H3: *β* = 0.402, *p <* 0.001), consistent with the view that EC serves as a motivational orientation that triggers ecological behavior (Hayat et al., 2025). This finding is consistent with COR theory’s investment principle: individuals with a sustained attitudinal-motivational orientation toward environmental protection are more likely to invest residual resources in behavioral engagement when the perceived cost of such investment is manageable (Hobfoll et al., 1990). Rather than attributing this to a congruence between behavior and an “internal resource system”—which would overextend COR’s original formulation—the alignment between EC and PEBs is understood here as reflecting the motivating force of a coherent attitudinal orientation that directs discretionary behavioral effort. In academic environments, particularly where autonomy and values are closely tied to professional identity, individuals tend to be highly concerned about environmental sustainability (Abo-Khalil, 2024).

The significant mediation effect (H4: *β* = 0.184, *p <* 0.001) identifies EC as an important psychological mechanism linking ILTs to PEBs. However, the mediation pattern is inconsistent rather than straightforward. While ILTs directly reduce PEBs by depleting employees’ resources, they also indirectly support PEBs by heightening EC. This suggests that faculty members exposed to illegitimate tasks may become more attentive to inefficiency, waste, and responsibility gaps, thereby strengthening their environmental concern. In turn, this heightened concern can motivate greater engagement in pro-environmental behaviors. Thus, EC partially counterbalances, rather than transmits, the negative association between ILTs and PEBs.

In academic settings, EC may therefore serve as a value-based orientation that helps explain why some faculty remain motivated toward sustainability despite unfavorable work conditions. Additionally, this mediation highlights EC’s significance as a resilience mechanism in reaction to HR practices that deplete resources and as a channel for the operationalization of employees’ internalized environmental values into tangible behaviors.

The dual moderating function of WOL significantly enhances the explanatory robustness of the model. WOL positively moderated the relationship between ILTs and EC (H5: *β* = 0.485, *p <* 0.001), suggesting that in conditions of elevated workload, the adverse implications of unsustainable human resource practices become increasingly pronounced, thereby motivating faculty to reaffirm their internal environmental principles as a means of coping. This observation is consistent with existing literature on role stress and ILTs, which posits that employees tend to respond to perceived organizational inequities by invoking personal values and intrinsic motivations as compensatory mechanisms (Ma and Xie, 2024; Zhao et al., 2023b). Furthermore, in the context of higher education, faculty members encountering administrative overload frequently reconceptualize stressors through a value-centric perspective, which may increase their concern about environmental issues (Abo-Khalil, 2024).

However, WOL exhibited a negative moderating effect on the relationship between EC and PEBs (H6: *β* = −0.368, *p* = 0.001), illustrating that resource depletion hampers the translation of concern into actionable behaviors, in line with research that underscores the intention-behavior gap under conditions of elevated job demands (Benedict, 2023). This trend corroborates earlier findings indicating that while psychological awareness and value-based concern may persist under duress, the actual realization of PEBs necessitates adequate cognitive and emotional resources, which are diminished in the face of substantial workloads (Stajkovic and Stajkovic, 2025; Szostek, 2021). In essence, workload serves as a boundary condition, demonstrating that the motivational significance of environmental concern does not invariably suffice to transcend structural and resource limitations.

### Theoretical contributions

This research presents four significant contributions to the theoretical framework surrounding sustainability behavior within organizational environments, particularly through the perspective of COR Theory (Hobfoll et al., 1990).

First, the results underscore the necessity of differentiating between cognitive and behavioral outcomes when implementing COR theory, thereby enhancing theoretical accuracy within sustainability scholarship. Although resource depletion has been persistently associated with diminished behavioral engagement (Demerouti, 2025), the findings of this study unveil a paradoxical phenomenon wherein depleted resources may concurrently incite cognitive responses such as increased EC. This illustrates that resource loss does not categorically inhibit all modalities of sustainability-related engagement. Such phenomena are especially pronounced in value-driven sectors such as academia, where moral, identity-related, or ethical obligations toward sustainability may be activated even amidst adverse organizational circumstances (Abo-Khalil, 2024; Hinduja et al., 2023). This distinction enriches COR theory by elucidating that cognitive activation can operate independently of behavioral enactment, thereby suggesting a more intricate process of resource allocation within multifaceted organizational frameworks.

Second, the inconsistent mediating role of EC enhances theoretical comprehension by clarifying indirect pathways through which unsustainable human resource practices affect PEBs. Specifically, EC partially counterbalances the negative direct association between ILTs and PEBs, indicating that the attitudinal pathway operates in the opposite direction from the behavioral depletion pathway. Importantly, this study advances COR-based theorizing by treating EC not as a resource per se, but as an attitudinal-motivational mechanism: a psychological orientation that can channel residual motivational energy toward sustainability even when broader resource conditions are constrained. This supports the conceptualization of environmental consciousness as a psychological orientation that facilitates PEBs even in resource-depleting contexts (Ding and Kuvaas, 2023; Hobfoll et al., 1990). By identifying EC as a mediating variable, this inquiry extends previous COR-focused research, which has predominantly emphasized direct impacts of stressors on behavior (Ding and Kuvaas, 2023), thus providing empirical support for the significance of internalized values and concern as a mechanism through which employees sustain sustainability outcomes in spite of organizational limitations.

Third, the divergent moderation effects of WOL illuminate the intricate boundary conditions under which sustainability behavior manifests. Specifically, WOL enhances cognitive responses while simultaneously constraining behavioral responses, thus offering empirical validation for the dual-path stressor interpretation within COR theory (Demerouti, 2025; Stajkovic and Stajkovic, 2025). This discovery advances existing scholarly discourse by demonstrating that organizational stressors can exert bifurcated effects, concurrently augmenting awareness while restricting enactment, thereby accentuating the necessity of considering both psychological and behavioral dimensions in the analysis of pro-environmental behavior.

Fourth, the positive association between illegitimate tasks and EC challenges established assumptions regarding resource depletion, revealing that unsustainable practices may elicit compensatory cognitive activation. When professional identity and environmental values are closely interlinked, employees may react to institutional neglect by reinforcing their internalized sustainability concerns (Yang et al., 2025). This revelation extends the discourse on identity-protective mechanisms, illustrating that cognitive activation can function as a compensatory reaction to organizational deficiencies, and offers a micro-level elucidation of how individual processes aggregate to influence broader sustainability trajectories, as underscored in co-evolutionary research (Hassan et al., 2024).

These contributions enhance theoretical understanding in three principal ways: they refine COR theory by distinguishing between cognitive and behavioral responses to resource depletion, identify environmental concern as a pivotal mediating mechanism, and underscore WOL as a boundary condition that influences the manifestation of sustainability behavior. This yields a more comprehensive and nuanced framework for elucidating how organizational practices, individual values, and resource dynamics interrelate to dictate pro-environmental behavior in higher education and other organizational contexts.

### Practical implications

The research findings provide actionable insights for academic administrators seeking to improve sustainability outcomes within the distinct framework of higher education institutions in Pakistan, where practices of unsustainable HRM and workload pressures are prevalent. By elucidating the manner in which micro-level resource dynamics influence organizational sustainability outcomes, this investigation underscores the pivotal role that academic leaders assume in connecting individual PEBs to expansive institutional systems.

The investigation uncovers a sustainability paradox wherein illegitimate tasks and excessive workloads may heighten environmental concern among faculty while concurrently diminishing their capacity to address these concerns effectively. Academic leaders within Pakistani universities can mitigate this issue by instituting workload management strategies, delineating roles, and legitimizing assigned responsibilities, thereby ensuring that faculty possess both the motivation and capability to participate in environmentally sustainable practices. In light of the challenges posed by public funding constraints and bureaucratic regulations, leaders must innovatively align sustainability objectives with institutional priorities and governmental policies, such as the guidelines established by the Higher Education Commission concerning campus sustainability and energy efficiency.

Environmental concern emerges as a critical mediating factor, indicating that ecological awareness may partially counterbalance, but cannot fully neutralize, the detrimental effects of unsustainable organizational conditions. Universities have the potential to institutionalize green training programs, workshops, and value-based campaigns that foster long-term ecological consciousness. Within the Pakistani context, this may involve the integration of sustainability modules into faculty development initiatives, the establishment of collaborations with local non-governmental organizations, or engagement in government-led projects such as the Green Campus Program.

The detrimental influence of unsustainable HRM on pro-environmental behavior emphasizes the significance of human resource practices in promoting sustainability. Academic leaders can alleviate these adverse effects by incorporating Green-HRM practices that advocate for participative decision-making, autonomy in role formulation, and recognition of environmentally responsible behavior. Such initiatives not only enhance faculty morale but also align with national directives that endorse the establishment of environmentally responsible universities.

Finally, the dual moderating role of workload suggests that sustainability initiatives ought to be strategically timed. During peak academic periods, universities may prioritize low-effort, high-visibility interventions, such as energy-efficient lighting, water conservation devices, or automated waste segregation systems. Conversely, more resource-intensive initiatives such as curriculum greening, research clusters focused on sustainability, or community outreach programs; should be allocated to periods of reduced workload, thereby ensuring faculty engagement and maximizing overall impact. By proactively addressing indicators of institutional strain, academic leaders can convert reactive environmental concern into sustained, coordinated action.

### Limitations and directions for future research

There are several constraints to this investigation that suggest possible areas for further inquiry. The cross-sectional framework inherent in the single-wave survey hinders the establishment of causal inferences in this study. While the results of this study are aligned with the COR theory, the possibility of reciprocal or lagged effects cannot be dismissed. Longitudinal or panel studies extending over an academic year could elucidate the dynamics by which mediation and moderation effects may augment, diminish, or even invert over time. The contextual parameters of this study’s dataset also limit the scope of generalizability. The sample is derived from private universities in Pakistan, a setting within an emerging economy characterized by unique workload conventions. Comparative examinations across public and private educational institutions, as well as Western and non-Western environments or resource-abundant and resource-scarce contexts, would contribute to a clearer understanding of boundary conditions and enhance external validity.

A mechanistic comprehension represents another domain ripe for future exploration. The positive association between illegitimate tasks and environmental concern implies the presence of identity-protective processes that qualitative methodologies might elucidate. Methodological approaches such as interviews or diary studies could disclose how faculty members transmute experiences of resource depletion into moral or environmentally conscious engagement. Further moderators warrant scholarly attention. Work overload appears to heighten environmental concern while simultaneously constraining its translation into proactive behavior, thereby affirming the dual-path stressor logic posited by COR. Individual characteristics (e.g., environmental self-efficacy), meso-level influences (e.g., departmental climate), and macro-level indicators (e.g., sustainability rankings) may additionally inform these effects. Multilevel modeling techniques could be employed to assess such cross-level contingencies.

Lastly, methodological pluralism has the potential to enrich insights into the micro-to-macro resource trajectory. In addition to surveys, system dynamics simulations and action research interventions can elucidate feedback mechanisms between HR practices, resource flows, and organizational sustainability outcomes. By addressing these limitations, forthcoming research can enhance the comprehension of how organizational practices either undermine or bolster collective capabilities for sustainability.

## Conclusion

This study demonstrates that sustainability in universities is influenced by a three-way interplay of organizational stressors, employees’ environmental concern, and their capacity to act. Applying the conservation of resources theory, this study results show that resource loss from ILTs and heavy workloads suppresses pro-environmental behavior, yet can also trigger a compensatory rise in environmental concern—especially in value-driven professions such as academia. Workload then determines whether that concern converts into action.

The tested mediation and moderation paths carry two actionable messages. First, green programmes must address “HR basics” (role clarity, fair task allocation, balanced workload) as seriously as awareness-raising. Second, interventions should separate cognition from behavior: universities can boost environmental concern through training and communication, but that concern will translate into pro-environmental behavior only when workload leaves employees with sufficient time and energy.

Instead of treating organizational stress and sustainability as incompatible, this study findings suggest that strategic approaches that recognize the cognitive-behavioral distinction and manage workload dynamics can create conditions where environmental awareness translates effectively into environmental action, even in challenging organizational contexts. Universities that do so will be better positioned to meet escalating expectations for environmental resilience.

## Data Availability

The original contributions presented in the study are included in the article/Supplementary material, further inquiries can be directed to the corresponding author.
