# Correction: Do illegitimate tasks undermine employees' pro-environmental behavior? Examining the roles of environmental concern and work overload

**DOI:** 10.3389/fpsyg.2026.2017857

**Published:** 2026-10-30

**Authors:** Abdullah Alromaihi, Muhammad Shahid Shams, Hanan Eid Badwy, Abdelaziz Ali Marzouk, Mohamed Adel Abdelrazek

**Affiliations:** 1 Department of Business Administration, College of Business and Economics, Qassim University, Buraidah, Qassim, Saudi Arabia; 2 Department of Business Administration, Abasyn University, Peshawar, Pakistan; 3 Surveying of Natural Resources in Environmental Systems Department, Environmental Studies & Research Institute, University of Sadat City, Menoufia, Egypt; 4 Business Administration Department, Faculty of Commerce, Kafr El-Sheikh University, Kafr El-Shaikh, Egypt; 5 Business Administration Department, Faculty of Commerce, University of Sadat City, Menoufia, Egypt; 6 Faculty of Media and Marketing, Midocean University, Moroni, Comoros

**Keywords:** environmental concern, higher education, illegitimate tasks, pro-environmental behavior, work overload, employment-based

In the published article, affiliation 3 (Surveying of Natural Resources in Environmental Systems Department, Environmental Studies & Research Institute, University of Sadat City, Menoufia, Egypt) was erroneously assigned to author Mohamed Adel Abdelrazek. This affiliation is now removed for author Mohamed Adel Abdelrazek. In addition, the affiliation “Faculty of Media and Marketing, Midocean University, Moroni, Comoros” was omitted for Mohamed Adel Abdelrazek.

The correct affiliations for author Mohamed Adel Abdelrazek are “Business Administration Department, Faculty of Commerce, University of Sadat City, Menoufia, Egypt” and “Faculty of Media and Marketing, Midocean University, Moroni, Comoros”.

The original version of this article has been updated.

